# Excessive levels of nitric oxide in rat model of Parkinson’s disease induced by rotenone

**DOI:** 10.3892/etm.2014.2099

**Published:** 2014-12-02

**Authors:** ZHONG-KUI XIONG, JUAN LANG, GANG XU, HAI-YU LI, YUN ZHANG, LEI WANG, YAO SU, AI-JING SUN

**Affiliations:** 1 Department of Radiotherapy, Shaoxing Second Hospital, Shaoxing, Zhejiang 312000, P.R. China; 2 Department of Radiotherapy, Shaoxing Campus, The First Affiliated Hospital, School of Medicine, Zhejiang University, Shaoxing, Zhejiang 312000, P.R. China; 3 Department of Clinical Medicine, Shaoxing University School of Medicine, Shaoxing, Zhejiang 312099, P.R. China; 4 Medical Research Center, Shaoxing People’s Hospital, Zhejiang University, Shaoxing, Zhejiang 312000, P.R. China; 5 Department of Radiotherapy, Jiangsu University Affiliated People’s Hospital, Zhenjiang, Jiangsu 212002, P.R. China; 6 Department of Laboratory Medicine, Shaoxing University School of Medicine, Shaoxing, Zhejiang 312099, P.R. China; 7 Department of Basic Medicine, Shaoxing University School of Medicine, Shaoxing, Zhejiang 312099, P.R. China; 8 Department of Pathology, Shaoxing People’s Hospital, Zhejiang University, Shaoxing, Zhejiang, Shaoxing, Zhejiang 312000, P.R. China

**Keywords:** nitric oxide, rotenone, Parkinson’s disease

## Abstract

Systemic rotenone models of Parkinson’s disease (PD) are highly reproducible and may provide evidence on the pathogenesis of PD. In the present study, male Sprague-Dawley rats (1-year-old) were subcutaneously administered with rotenone (1.5 mg/kg/day) for six days and observed for the following three weeks. Compared with the control rats, a significant decrease was observed in the body weight and a marked increase was observed in the areas under the behavioral scoring curves in the rotenone-treated rats. Immunohistochemical staining revealed that the abundance of nigral tyrosine hydroxylase (TH)-positive neurons was markedly reduced following rotenone treatment. ELISA and neurochemical assays demonstrated a significant increase in the levels of nitric oxide (NO) and NO synthase, whereas a marked decrease was observed in the thiol levels in the brains of the rotenone-treated rats. Thus, subacute rotenone treatment was found to induce behavioral deficits and the loss of nigral TH-positive neurons which may be associated with the excessive levels of NO in the rat brains.

## Introduction

Parkinson’s disease (PD) is a common neurodegenerative disease, characterized by the selective degeneration and loss of nigral dopaminergic neurons leading to striatal dopamine (DA) depletion (1,2). Typical symptoms include the loss of ~50% of dopaminergic neurons in the substantia nigra and >70–80% loss of DA in the striatum (1,3,4). PD is mainly regarded as a disease of aging (5), affecting 1–2% of the world’s population aged ≥60 years and almost 4% of individuals aged >85 years (6,7).

Increasing evidence has indicated that excessive nitric oxide (NO) production contributes to the aging and PD development processes (8,9). Aging has been shown to alter the brain arginine metabolism of male Sprague-Dawley (SD) rats (10). In addition, a previous study has demonstrated that the mRNA and protein expression of neuronal NO synthase (NOS) is age-dependent in the brain cortex of rats (11). Higher levels of neuronal NOS (nNOS) and inducible NOS (iNOS) were observed in the substantia nigra of PD patients and animal models (12,13). Furthermore, genes coding for NOS have been shown to generate excess NO, contributing to neurodegeneration in PD (12). The iNOS expression increase has been shown to be inversely correlated with the immunolabeling of tyrosine hydroxylase (TH), a marker of DA neurons. A previous study has demonstrated that animals pretreated with the NOS inhibitor, N(G)-nitro-L-arginine methyl ester, exhibited complete protection against amphetamine-induced body rotations (14).

Epidemiological evidence has indicated that pesticides and other environmental exposures may play a role in the development of idiopathic PD (15,16). Rotenone is a natural, plant-derived pesticide. A previous study demonstrated that exposure to rotenone was associated with an increased risk of PD development in the study population (17). In addition, NOS genes may interact with each other or with environmental factors in PD (12). Chronic rotenone administration has been shown to lead to significant injury of the nigrostriatal system, which is mediated by increased NO generation (18,19). Compared with young rats, middle-aged rats (12–14 months old) have been shown to be more sensitive to rotenone (20). However, the levels of NO in the brain tissue of middle-aged rats that were subacutely induced by rotenone have been rarely reported.

In the present study, a subacute rotenone-induced rat model was established using middle-aged SD rats to investigate the effect of rotenone on NO production in the brain.

## Materials and methods

### Animals and rotenone treatment

All the experimental procedures were approved by the Animal Care and Use Committee of Shaoxing University School of Medicine (Shaoxing, China). Male SD rats (n=25; 1-year-old) were purchased from the Zhejiang Provincial Experimental Animal Center (Hangzhou, China). The rats were pair-housed in an environmentally controlled facility (12/12 h light/dark cycle; temperature, 22±2°C; relative humidity, 50±5%) and were provided with food and water *ad libitum*. The rats were randomly divided into two groups, including the control (n=9) and rotenone-treated (n=16) groups.

Prior to the experimental procedure, the rats were acclimatized for one week. Rotenone (Sigma-Aldrich, St. Louis, MO, USA) was dissolved in dimethyl sulfoxide to prepare a solution with concentration of 50 mg/ml, which was further diluted to 2 mg/ml using rapeseed oil. Rats in the rotenone-treated group were subcutaneously injected with 1.5 mg/kg/day rotenone solution for six days. Rats in the control group were injected with the same solution dose, without rotenone.

### Behavioral study

The behavioral scoring method described by Chen *et al* was used to daily assess behavioral alterations in the rats between the day prior to rotenone treatment and three weeks after termination of the treatment (total 27 days) (21). The rat behavioral scoring was as follows: 0, no symptoms; 1, dirty and bristling fur, bending of the back, attenuation of resisting arrest or decrease in passive movement; 2, significant decrease in passive movement, bradykinesia, tremors or unsteady movement; 4, inability to move in a straight line or unilateral rotation; 6, unilateral lying posture or paralysis, walking or eating difficulties; 8, complete unilateral paralysis, spasms, marked weight loss or inability to eat; 10, near-mortality or mortality.

### TH immunohistochemical examination

After three weeks of observation following treatment completion, the rats were intraperitoneally anesthetized with pentobarbital sodium (50 mg/kg body weight) and sacrificed by cervical dislocation. The rats were intracardially perfused with 30 ml 0.01 M phosphate-buffered saline (PBS; pH 7.4), followed by 200 ml 4% formaldehyde in PBS. The rat brains were dissected and postfixed in 4% formaldehyde in PBS at room temperature for 2 h. Next, the brains were cryoprotected in 20% sucrose in PBS at 4°C overnight, embedded in paraffin and cut into 25-μm coronal sections using a microtome (Leica Microsystems Nussloch GmbH, Nussloch, Germany). Prior to staining, the sections were dewaxed by washing three times with xylene for 15 min and infused in 100, 95 and 75% alcohol, followed by water (5 min each time).

The sections were treated with 0.1% Triton X-100 (Aladdin Industrial Corporation, Shanghai, China) and 3.0% H_2_O_2_ in 0.01 M PBS (pH 7.4) for 10 min. Subsequently, the sections were treated with 3% bovine serum albumin (Amresco, Solon, OH, USA) in PBS for 15 min to reduce nonspecific antibody binding. Next, the samples were incubated with rabbit anti-TH polyclonal antibody (1:100; Millipore, Billerica, MA, USA) in PBS containing 3% bovine serum albumin at 4°C overnight and 37°C for 30 min. The sections were washed with PBS and incubated with biotinylated sheep anti-rabbit secondary immunoglobylin G (1:10; EarthOx, LLC, San Francisco, CA, USA) for 1 h at room temperature, followed by incubation with streptavidin-biotin complex (1:10; Boster Bio-Engineering, Co., Ltd., Wuhan, China) in PBS (pH 7.4) for 30 min. Next, immunolabeling was visualized using 0.05% 3,3′-diaminobenzidine (Boster Bio-Engineering, Co., Ltd.)/0.005% H_2_O_2_ in Tris-buffered saline and rinsed in water to terminate the reaction. Finally, the sections were dehydrated, cleared and sealed for image capture. Images were captured with an Eclipse TS100 microscope (Nikon Corporation, Tokyo, Japan)

### Preparation of brain homogenate supernatant

Half of the brain tissue from each rat, with the exception of the cerebellum, was weighed and homogenized in 9 volumes of normal saline in an ice bath for 8 min (3 sec on and 5 sec off) using a JY92-IIDN ultrasonic cell crusher (Ningbo Scientz Biotechnology Co., Ltd., Ningbo, China). The samples were subsequently centrifuged at 2,556 × g for 10 min at 4°C. The total concentration of supernatant proteins was determined using a BCA Protein Assay kit (Pierce Biotechnology, Inc., Rockford, IL, USA). The supernatants were stored at −80°C for subsequent ELISA and neurochemical analyses.

### ELISA analysis

ELISA kits (RapidBio, West Hills, CA, USA) were used to determine the levels of NOS and superoxide radicals in the supernatants of the rat brain homogenates, according to the manufacturer’s instructions. A total of 50 μl samples or reference standards were pipetted into the microplate wells and incubated for 10 min at room temperature. An enzyme-linked antibody (EarthOx Life Sciences, Millbrae, CA, USA) specific to NOS or superoxide radicals was added to the wells and incubated for a further 2 h in order to bind to NOS or superoxide radical ligand. A substrate solution consisting of H_2_O_2_ and tetramethyl benzidine was added to the samples and the intensity at 450 nm was measured using an automicroplate reader (Model 2010; Anthos Labtec Instruments, GmbH, Salzburg, Austria). A YJZXM008 autowasher (Anthos Labtec Instruments, GmbH) was used to wash the microplate wells. Each sample was assessed in duplicate.

### Neurochemical analysis

The activity of superoxide dismutase (SOD) in the supernatants of brain homogenates was determined using a colorimetric total mercapto(-SH) measurement kit. assay kit (Nanjing Jiancheng Bioengineering Institute, Nanjing, China), according to the manufacturer’s instructions. Briefly, the supernatants and reagents were mixed and then incubated at 37°C for 40 min, followed by 10 min of chromogenic reaction. SOD activity was determined using a spectrophotometric method (22) to measure the absorbance at 550 nm. The results are expressed as units of SOD activity units/mg protein, where one unit of SOD activity is the amount of enzyme required to exhibit 50% dismutation of the superoxide radical.

The activity of peroxidase (POD) was determined in the supernatants of the rat brain homogenates using a colorimetric assay kit (Nanjing Jiancheng Bioengineering Institute), according to the manufacturer’s instructions. In brief, the supernatants and reagents were vortexed for 10 sec and placed in a water bath at 37°C for 30 min. A color-developing agent (from the ELISA kit, RapidBio) was subsequently added and left to react for 1 min. The absorbance at 420 nm was measured using an automicroplate reader.

The levels of thiols in the supernatants of the rat brain homogenates were detected using a total mercapto (-SH) measurement kit (Nanjing Jiancheng Bioengineering Institute), according to the manufacturer’s instructions. In the presence of thiol compounds, colorless 5-5′-dithiobis(2-nitrobenzoic acid) (DTNB) is converted to yellow 5-mercapto-2-nitrobenzoic acid. The absorption spectrum of DTNB does not interfere with thiol detection (23). A total of 10 μl of sample, standard or blank were reacted with working solutions for 5 min at room temperature. The optical densities of the products at 405 nm were detected using an automicroplate reader.

The levels of NO in the supernatants of the rat brain homogenates were determined using a commercial kit (Nanjing Jiancheng Bioengineering Institute), according to the manufacturer’s instructions. NO is a highly reactive gas that is easily transformed into NO_2_^−^ and NO_3_^−^ *in vivo*, while NO_2_^−^ is finally transformed into NO_3_^−^ and NO_3_^−^ is reduced to NO_2_^−^ by nitrate reductase. The concentration of NO_2_^−^ determined by the spectrophotometric method was used to determine the total levels of NO at 550 nm.

### Statistical analysis

Statistical analysis was performed using the SPSS 13.0 software (SPSS, Inc., Chicago, IL, USA). The data are presented as the mean ± standard error of mean. The differences among the variables of the different groups were evaluated by one-way analysis of variance followed by a post hoc Student-Newmann-Keuls test. Student’s t-test was used when only two sets of data were compared, while χ^2^ and Fisher’s exact tests were used in the comparison of categorical data as required. P<0.05 was considered to indicate a statistically significant difference.

## Results

### General results

**Figure 1:**
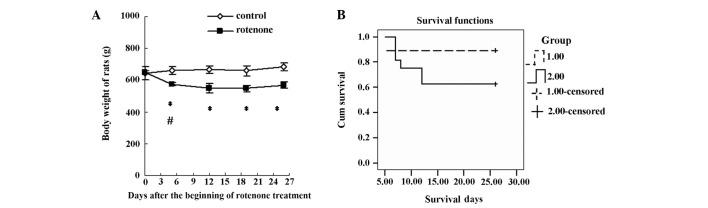
(A) Effect of rotenone on the body weight of rats prior to and at weeks 0, 1, 2 and 3 following rotenone treatment (mean ± standard error of the mean). (B) Survival rates of the rotenone-treated and control rats. The control and rotenone-treated groups consisted of 9 and 16 rats, respectively, at the beginning of the experimental procedure and 8 and 10 rats, respectively, at the end of the experimental procedure. ^*^P<0.05, vs. control group; ^#^P<0.05, vs. rats on day 0. Group 1, control; group 2, rotenone-treated group.

Gastrointestinal disorders, particularly severe constipation and delayed gastric emptying, are core symptoms of PD that affect the majority of patients. Chronic rotenone exposure has been found to reproduce PD gastrointestinal neuropathology (24). The present study observed that rats in the rotenone-treated group consumed less food and water compared with rats in the control group. As shown in Fig. 1A, the body weight of rats markedly decreased following rotenone treatment for six days compared with the control group. In addition, a significant decrease was observed in the body weight of rotenone-treated rats at weeks 0, 1, 2 and 3 following rotenone treatment compared with the control group rats.

During the experimental procedure, one rat succumbed in the control group at day 5 following the beginning of the experiment. By contrast, as shown in Fig 1B, six rats succumbed in the rotenone-treated group (three, one and two rats succumbed at day 7, 8 and 12, respectively, after the initiation of the rotenone treatment). No differences were observed in the survival rate among the control and rotenone-treated groups (χ^2^=1.552; P=0.213), which is partially attributable to the small sample size.

### Behavioral rating scale

**Figure 2:**
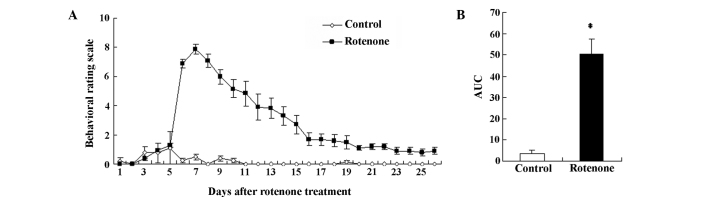
Effect of rotenone on the behavioral performance of the experimental rats. (A) Behavioral scores. (B) The AUC of the behavioral rating scale was calculated in each group. The data are expressed as the mean ± standard error of the mean. ^*^P<0.05, vs. control group. AUC, area under the curve.

As shown in Fig. 2A, the behavioral scores of rotenone-treated rats were found to be significantly increased following the administration of rotenone when compared with the control rats. The rotenone-treated group scores reached a peak on day 7. The area under curve (AUC) of the behavioral rating scale of each rat was calculated. The differences between the AUCs of behavioral rating scales in rotenone-treated and control rats were analyzed statistically. As shown in Fig. 2B, a significant increase was observed in the area under the behavioral rating scale curves of the rotenone-treated rats compared with the control rats. Therefore, rotenone treatment may induce marked behavioral deficits mimicking the movement disorder of PD.

### Nigral TH-positive neurons in the rat brains

**Figure 3:**
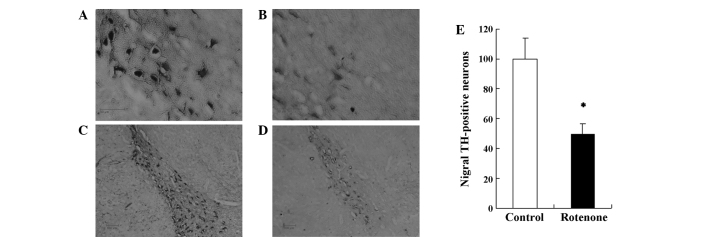
Effect of rotenone on the number of nigral TH-positive neurons. TH-immunoreactive staining at ×400 magnification in the (A) control and (B) rotenone-treated rats (scale bar=50 μm) and ×100 magnification in the (C) control and (D) rotenone-treated rats (scale bar=100 μm). (E) Relative number of nigral TH-positive neurons in each group (n=3 in each group). Two homologous sections from each rat with two sides of the substantia nigra were investigated at ×100 magnification to determine the number of nigral TH-positive neurons. ^*^P<0.05, vs. control group. TH, tyrosine hydroxylase.

PD is characterized by the loss of ~50–70% of dopaminergic neurons in the substantia nigra pars compacta. TH is a rate-limiting enzyme in the catalyzed synthesis of dopamine and a transmitter and marker of dopaminergic neurons. The present study observed the effects of rotenone on the number of nigral TH-positive neurons in rat brains. As shown in Fig. 3, the number of nigral TH-positive neurons were found to be decreased in the rotenone-treated rats (49.4±7.3%) when compared with the control rats (100.0±14.2%). Therefore, the number of nigral TH-positive neurons in the rotenone-treated rats was decreased by 50.6% when compared with the control rats.

### Markers of oxidative, nitrosative and nitrative stress

**Figure 4:**
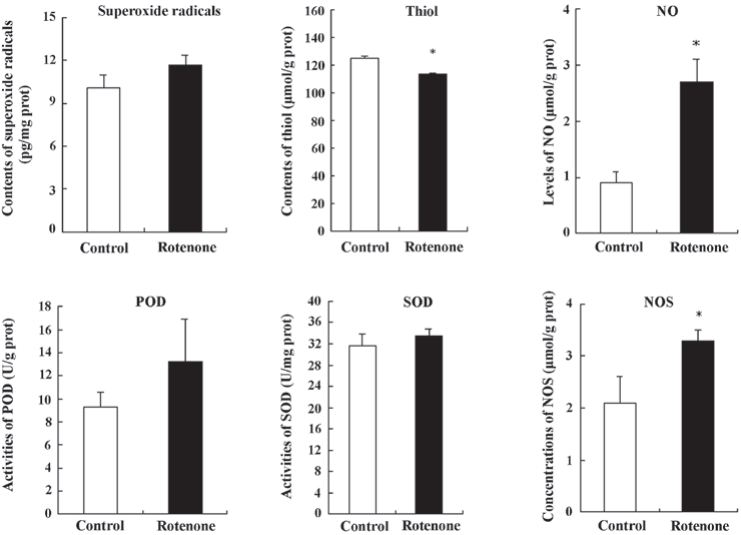
Effect of rotenone on oxidative, nitrosative and nitrative stress in the rat brains. Three control and six rotenone-treated rats were used to determine the levels of superoxide radicals and NO and the activities of POD, SOD and NOS, while three control and five rotenone-treated rats were used to determine the thiol levels. ^*^P<0.05, vs. control group. NO, nitric oxide; POD, peroxidase; SOD, superoxide dismutase; NOS, NO synthase.

Previous studies have demonstrated the major contribution of reactive oxygen species, in particular NO and hydroxyl radicals, to the pathophysiology of PD (25–27). The effect of rotenone on oxidative, nitrosative and nitrative stress markers in rat brains was determined in the current study. As shown in Fig. 4, the brain thiol levels of rotenone-treated rats (113.6±0.8 μmol/g protein)were found to be decreased by 10.0% compared with the control rats (125.0±1.4 μmol/g protein). By contrast, the brain NO levels of rotenone-treated rats (2.7±0.4 μmol/g protein) were found to be increased by 200% compared with the control rats (0.9±0.2 μmol/g protein). In addition, a 57.1% increase was observed in the brain NOS concentration levels of rotenone-treated rats (3.3±0.2 μmol/g protein) compared with the control rats (2.1±0.5 μmol/g protein). No statistically significant differences were observed in the levels of superoxide radicals (10.1±0.9 vs. 11.7±0.7 pg/mg protein), POD activity (9.3±1.3 vs. 13.2±3.7 U/g protein) and SOD activity (31.7±2.1 vs. 33.4±1.4 U/mg protein) between the control and rotenone-treated rats, respectively.

## Discussion

Rotenone, a herbicide and insecticide, is the most potent member of the rotenoid family of neurotoxins that are found in tropical plants in nature. Increasing evidence has indicated that rotenone exposure is associated with an increased risk of developing PD (28). The oxidation of DA, a transmitter of dopaminergic neurons, facilitates rotenone-dependent neurotoxicity in rat substantia nigral dopaminergic neurons (29). The differences in DA concentration following intoxication, with regards to age and gender, may be due to the increased susceptibility of males and older animals to the toxic effects of neurotoxin and the aggravated process of recovery in older brains (30). Rotenone inhibits the expression of TH, which influences the synthesis of DA in PC12 cells through the activation of plasma membrane adenosine triphosphate-sensitive potassium channels (31). However, chronic rotenone treatment has been shown to induce behavioral effects in mice with no pathological signs of Parkinsonism (32). The results of the present study revealed that 50.6% of the nigral TH-positive neurons were lost and behavioral deficits were apparent in middle-aged rats that were subacutely induced by rotenone.

Rotenone not only causes damage to nigrostriatal dopaminergic neurons, but also induces α-synuclein aggregation and Lewy body-like formation, which are known changes in the pathology of PD (20,33,34). Drolet *et al* demonstrated that a circumscribed exposure to environmental toxicants may lead to the delayed appearance of Parkinsonian α-synuclein pathology in the enteric nervous system and an associated functional deficit in gastrointestinal motility (24). The present study observed a rapid weight loss in rats following rotenone administration, which may be associated with gastrointestinal dysfunction (24). Pan-Montojo *et al* demonstrated that rotenone may promote the release of α-synuclein by enteric neurons (35). The released enteric α-synuclein is then absorbed by presynaptic sympathetic neurites and retrogradely transported and accumulated at the soma. These authors hypothesized that pesticides may initiate the progression of PD pathology, which is associated with the transneuronal and retrograde axonal transport of α-synuclein (35).

A previous study has indicated that NOS activation and peroxynitrite ion overproduction may be involved in the etiopathogenesis of PD (36). Levecque *et al* observed an association between PD and polymorphisms in the nNOS and iNOS genes in a community-based case-control study (37). Hancock *et al* showed that the interaction of NOS1 and NOS2A, the genetic risk factors for PD, with established environmental factors (including pesticides) may modulate the effect of these factors on the pathogenesis of PD (12). However, Huerta *et al* found no association among PD and three polymorphisms in the eNOS, nNOS and iNOS genes (38). Furthermore, significantly low circulating levels of NO and reduced salivary NOS were demonstrated in patients with PD (39,40). Chronic administration of rotenone was found to increase the NO levels in the cortex and striatum of rats, leading to significant injury in the nigrostriatal pathway (18,19). The experimental results of the present study revealed a significant increase (57.1%) in the concentration of the NOS protein and a marked increase (200.0%) in the levels of NO in the brain homogenates of rats that were subacutely treated with rotenone, compared with the control rats. Overproduction of NO has been shown to result in neuronal damage and may be associated with S-nitrosylation or nitration of certain important proteins, including the S-nitrosylation of Parkin (41,42), protein-disulphide isomerase (43), mitochondrial complex I (44), peroxiredoxin-2 (45) and the nitration of α-synuclein (46) in the neurodegenerative process of PD.

A previous study has reported that the critical role of glutathione (GSH) is to maintain functional mitochondria (47). Depletion in the levels of the thiol reducing agents, GSH and GSH disulfide, is the earliest reported biochemical event occurring in the Parkinsonian substantia nigra prior to the selective loss of complex I activity associated with the disease, which is believed to contribute to subsequent dopaminergic cell death (48). The present study demonstrated a significant decrease in the levels of thiol in rotenone-induced rat brains, which is in accordance with the results observed by Di Monte *et al* (49).

In conclusion, the overactivation of NOS and subsequent excessive levels of NO in rat brains may play an important role in the behavioral deficits and loss of nigral TH-positive neurons observed in the subacute rotenone-induced rat model of PD.
